# A Description of Infection Control Structure in Primary Dental Health Care, Brazil

**DOI:** 10.1155/2021/5369133

**Published:** 2021-07-30

**Authors:** Emílio Prado da Fonseca, Edmilson Antônio Pereira-Junior, Andréa Clemente Palmier, Mauro Henrique Nogueira Guimarães Abreu

**Affiliations:** ^1^Department of Community and Preventive Dentistry, School of Dentistry, Universidade Federal de Minas Gerais, Belo Horizonte, Minas Gerais, Brazil; ^2^Faculty of Education, Universidade Federal de Minas Gerais, Belo Horizonte, Minas Gerais, Brazil

## Abstract

**Objective:**

This study is aimed at describing a score to assess infection control structures in Oral Health Teams (OHT) in Primary Health Care (PHC) in Brazil.

**Methods:**

Secondary data from a national external evaluation of PHC conducted in 2017 and 2018 were analyzed. The construction of the score used 14 variables, divided into the following: structural characteristics of the PHC, infection control equipment under conditions of use, and biosafety supplies in sufficient quantity. The questions were mostly dichotomous (yes/no). Descriptive analyses were carried out to characterize the OHT and factor analyses to reduce the number of observed variables to a specific number of factors.

**Results:**

Among 20,301 health units with OHT, 4,510 (22.2%) units did not have washable floors, ceilings, and walls; 8,406 (41.4%) did not have a sealer; 16,780 (82.7%) did not have taps with noncontact activation, and 4,663 (23.0%) units did not have rubber gloves. Regarding personal protective equipment (PPE), 1,618 (8.0%) units did not have a sufficient quantity of basic PPE. Three factors were defined to explain the 14 evaluated variables. The South region had the best score of infection control, while the North had the worst.

**Conclusions:**

Regional inequalities in the failures in infection control structures identified in PHC with OHT were related to the physical structure, equipment, and supplies used for infection control and the absence of PPE for OHT.

## 1. Introduction

The Brazilian Ministry of Health introduced the monitoring and evaluation of PHC through the National Program for Improving Access and Quality of Primary Care (PMAQ-AB). For this, it proposes a set of qualifications, monitoring, and evaluation strategies for Primary Health Care (PHC) units, together with Oral Health Teams (OHT). OHT are composed by one dentist and one or two dental assistants. OHT provide community actions and surgical, restorative, preventive procedures at PHC level [1]. Each PHC unit is evaluated and monitored based on the observation of the facilities, health indicators, and interviews with service users and human resources at PHC. Brazilian Ministry of Health used a formula that qualified each PHC team based on an overall score that is a weighted average on their participation in the self-assessment activities, health indicators, adequate use of electronic health records, and a group of indicators from the external evaluation [2]. A previous study with 16,202 OHT, evaluated by the second cycle of the PMAQ (2013-2014), identified that only 208 (1.3%) teams performed all 12 infection control structures and that 1,078 units had worse structures for infection control [3].

Furthermore, infection control is an important stage in the structure, work process, and health security for public services offered to the population [4]. Review study addresses the issue of infection control and prevention in oral health services implemented around the world [4]. The results show wide variation in infection control procedures, despite the fact that the recommendations for infection control are the same in all locations, mainly due to the new scenario provided by the COVID-19 (SARS-CoV-2) pandemic, which rekindled the worldwide discussion on infection control in dentistry [4, 5]. Thus, oral health care presents a high risk of contamination and spread of COVID-19, due to the production of aerosols, proximity to a patient's face, and interpersonal contact, as well as exposure to saliva, droplets, blood, equipment surfaces, furniture, objects, and clothing [6].

In that sense, it is possible to maintain gaps in infection control in the PHC units with OHT and it is important to define a specific score to measure infection control in public dental services. Hence, this study is aimed at describing a score to assess infection control structures in Oral Health Teams (OHT) in PHC in Brazil.

## 2. Materials and Methods

### 2.1. Study Design

This is a health evaluation study based on secondary data from a national scope—the external evaluation of PHC units with OHT in Brazil that participated in the third cycle of the PMAQ-AB in 2017 and 2018. The third cycle of PMAQ-AB was developed by the Ministry of Health in partnership with several teaching and research institutions in the country [7]. The external evaluation of the PMAQ-AB consists of collecting information to analyze the conditions of access to and quality of PHC units. It seeks to recognize and value the efforts and results of the OHT and municipal health managers in qualifying PHC [8]. The external evaluation instrument is organized into four modules, according to the data collection method. The external evaluation instrument consisted of 903 questions related to quality standards [8].

This third cycle of PMAQ dataset has information from 22,046 dental offices (Table 1) (URL: https://sage.saude.gov.br/) (7).

### 2.2. Study Variables

The development of Infection Control Structure Score (ICSS) used 14 variables that analyzed the structural/physical conditions of dental offices, which were described in Table 1. No missing data was identified for these 14 variables.

### 2.3. Statistical Analysis

The ICSS was measured by 14 variables related to the existence of equipment and physical structure of the health unit facility. The metric was developed using the factor analysis statistical technique, which is aimed at describing the original variability of a random vector, in terms of a smaller number of random variables related to the original vector through a linear model [9].

The factor analysis model adequacy was verified using the Kaiser-Meyer-Olkin (KMO) statistic, which provides the model's adjustment coefficient to the dataset [9]. Values of this measure in the 0.5 range would not be desirable and would require correction measures in the data samples through the exclusion or inclusion of new variables. Coefficients higher than 0.7 could be considered adequate, while coefficients upper than 0.9 would be considered excellent [9]. The Bartlett sphericity test used in this study verifies whether or not the variables are correlated with each other and corroborates the model's fit quality [9]. The component values will be used as a weighting factor in the operationalization of the ICSS, according to the following equation:

(1) $$\begin{array}{c} \text{ICSS}=\frac{\left[\left(\text{value}\times \text{good ventilation }\right)\right.+\left(\text{value}\times \text{floor with washable}\right)+\left(\text{value}\times \text{mold near sink }\right)+\cdots .+\left(\text{value}\times \text{PPE score}\right)}{\text{Sum of factorial loads}} \end{array}$$

The number of factors was defined to represent the entire set of variables and verify the fit measures of the model [9]. The allocation of each variable to the respective factor was then performed [9]. It is necessary to determine the number of factors to be used in the explanation of the infection control structure. This finding is based on the number of components with eigenvalues greater than one and the verification of the jump point obtained by the eigenvalues.

Descriptive analyses were carried out using Excel for Windows. The factor analysis was performed in the computer program factor [10]. ICSS values were described by type of PHC unit. The types of PHC unit were health center, i.e., a facility that offers health care to a population, in a programmed way or not, by technical professional, with presence or not of the physician; basic health unit, i.e., a facility that offers basic and comprehensive care to a population, in a programmed way or not, in basic medical specialties, which is able to offer dental care and other health professionals; mixed health unit, i.e., a facility that provides basic and comprehensive health care, programmed or not, in basic medical specialties, which is able to offer dental care and other professionals, with a hospitalization unit, under a single administration; others, which included, specialty center, polyclinic, and hospitals. The results were also stratified by the Brazilian geographic regions (North, Northeast, Center, Southeast, and South).

### 2.4. Ethical Issues

The study analyzed the public database of the Brazilian Ministry of Health. No subject or health team will be identified. The project was approved by the Ministry of Health through an Ethics Committee on Research involving human beings (CAAE 77847417.9.0000.0055 and approval number 2346623).

## 3. Results

The descriptive analysis of the physical structure of the 20,301 health units with OHT is shown on Table 1.

The variables “clogged sink,” “closed off sink,” and “presence of hydraulic and sanitary tubing” were excluded from the factor analysis due to the fact that they are variables that almost all health units (≥99%) had in good working condition.

**Figure 1:**
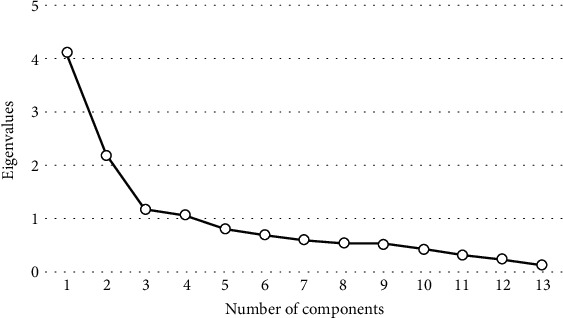
Screeplot of factorial analysis on the Infection Control Structure Score (ICSS), Brazil, 2017-2018.

Three factors were defined to explain the 14 analyzed variables. The three components presented eigenvalues of greater than one, while the third factor represented the jump point (Figure 1). Regarding the model's adequacy, Bartlett's sphericity test proved to be significant (*p* < 0.001), and the KMO statistic was equivalent to 0.712.

The factorial analysis identified relationships among the study variables and summarized a set of factors. The infection control variables were grouped into 3 factors. Factor 1 was formed by the following variables: presence of mold, tap without running water, pungent sewage smell, and lack of water; factor 2: good ventilation and washable floors and walls; factor 3: presence of autoclave, sealers, waste bins, rubber gloves, cleaning materials, packaging material, and PPE (Table 2).

The formula with the values for each component with the respective values used as weighting factors that enabled the ICSS operation was the mean ICSS value was 0.902 (SD = 0.109), ranging from 0.090 to 1.000. The median value was 0.934.

(2) $$\begin{array}{c} \text{ICSS}=\frac{\left[\left(0.510\times \text{good ventilation}\right)\right.+\left(0.517\times \text{floor washable}\right)+\left(0.572\times \text{mold near sink}\right)+\cdots .+\left(0.482\times \text{PPE score}\right)}{8.084}. \end{array}$$

**Figure 2:**
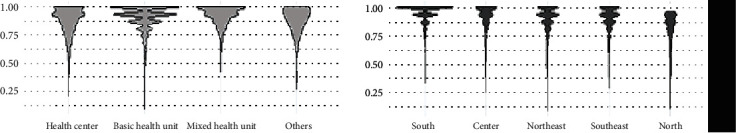
Distribution of Infection Control Structure Score (ICSS) according to the type of PHC unit and Brazilian geographic region, Brazil 2017-2018.

The South region, followed by the Center region of the country, presented the best averages for the ICSS. On the other hand, the North region presented the worst average for the ICSS. A variation was also observed in the infection control structure among the unit types, with the mixed-type unit presenting the best ICSS (Figure 2).

## 4. Discussion

The study synthesized 14 different characteristics about the physical structure for dental infection control in Brazilian PHC in 2017-2018. Factor analysis evaluated the structural aspects of infection control in dental settings, with no loss of quality information [11]. It was shown that the three factors identified in the study were able to explain the original variables and highlight the permanence of failures and regional inequalities in infection control in PHC public dental clinics among Brazilian regions.

Factors 1 and 2 represent the physical structure of the basic units, and factor 3 represents the biosafety equipment and supplies necessary to carry out infection control. The variables “Faucet without water” and “packaging material” had the highest factor loadings. Water is considered a critical input for hand sanitization and the cleaning and washing of the instruments. The Centers for Disease Control and Prevention (CDC), along with infection control experts from other federal agencies, scientists, private organizations, and professionals, published Guidelines for Infection Control in Dental Healthcare Settings [12]. New evidence suggested the importance of maintaining the quality of water in the dental unit, through its supply system and its monitoring in the last 12 months [12]. In addition, this study identified units without material for packaging dental instruments for sterilization. The “packaging material” is an essential wrapping material to cover, pack, maintain the sterilization of dental instruments, and ensure the protection of the instruments from possible contamination caused by the environment and improper handling, preserving the sterility of these materials until they are used [13]. The steps involved in steam sterilization, including packaging, should follow standard procedures to ensure the recommended level of sterility of the instruments [13]. Moreover, the management and support required to ensure the sterility of dental instruments can occur outside the healthcare service in a central unit [1]. The focus of this study was to discuss infection control practice safety to produce a reduction in disease transmission [13].

The present study identified a lack of biosafety supplies and PPE in sufficient quantity for oral health professionals. In this sense, health care units with low ICSS may lack biosafety supplies and PPE with serious implications upon infection control. Infection control programs consider biosafety supplies and PPE as important safety barriers for the most vulnerable areas of the body, such as mucous membranes (eyes), face, skin, and hands of the oral health professionals, as well as for disease transmission [4]. PPE, including protective clothing, masks, glasses, and disposable gloves, should be worn during any clinical contact, regardless of the patient's health history [4]. One previous study showed that there is a variation in the use of PPE by oral health professionals and that rubber gloves are the most used [4]. However, the quality of gloves and the possibility of exposure, especially in procedures with a higher risk of exposure (surgeries), are challenges for infection control [4]. It is noteworthy that the PPE evaluated in the last cycle of PMAQ-AB did not include new PPE required in times of the COVID-19 pandemic [14, 15]. Equipment, such as face shields, disposable aprons, and N95 masks, are now included in a new work routine and infection control that will challenge all dental services in both the world and the OHT in PHC in Brazil [14, 15]. Previous studies identified differences between macroregions in the process of dental infection control in public services [16]. In this study, the South region had the best infection control structure, while the North region had the worst structure, corroborating previous national findings [16]. Studies show that Brazil is a country marked by regional socioeconomic inequalities that impact health [7, 17] and that the country's health policies should be reoriented to minimize such inequities [17]. In oral health, the social level is a determining factor of both the organization of services and epidemiological conditions in various populations of different countries [18, 19]. Public efforts at national, state, and municipal levels should be made to improve the infection control framework. This improvement has the potential to impact oral health work processes, as well as improve the health status of individuals and the population [16]. This study showed an improvement of 11.8% in the item, good ventilation, when compared to the 2013-2014 evaluation among OHT in PHC in the state of São Paulo [16]. Compared to a previous study with national data from 2013 to 2014 [1], an improvement of 9.9% was found, which is very positive. Dental care generates a significant amount of aerosols that can be contaminated with microorganisms, especially SARS-CoV-2, the virus responsible for COVID-19, and a ventilation system, whether natural or mechanical, can reduce the concentration of infectious aerosols [3–6]. Moreover, it is essential that health care facilities have safe and appropriate conditions, including the availability of treated water, sanitation, hygiene, energy, waste management, and PPE to promote the health of patients and staff [20]. A previous study from health facilities in seventy-eight low- and middle-income countries estimated that 50% of the facilities have no piped water on site, 33% do not have adequate sanitation facilities, 39% do not have soap for handwashing, 39% do not have proper disposal for infectious waste, 73% do not have sterilization equipment, and 59% do not have reliable electricity [20]. Only 2% of the facilities provide all four services of water, sanitation, hygiene, and waste management [20]. Inadequate structural conditions contribute to the spread of infections in health care facilities through contaminated water, hands, fomites, food, equipment, sharps, and incorrect disposal of infectious waste [20]. The current infection control precautions should adopt strategies for care in external areas; the limiting of physical contact; the separation of care areas, waiting distance, and physical barriers; the availability and rational use of PPE; and teleconsultation in order to maintain of care [21].

With the increase in investments and the growing expansion of the supply of public dental services through the expansion of OHT in PHC, the access to public oral health services, equipment, and dental procedures has increased; consequently, the challenge of physical structure, cleaning, and sterilization of instruments used in dental care has also increased [4, 14, 22]. Cleaning the instruments is quite relevant prior to sterilization to diminish the number of microorganism and also the visible biological tissues, body fluids, and dental materials. Sterilized instruments should be also stored in dry, covered, or closed cabinets [23]. All these last items could be evaluated in a next monitoring of PHC in Brazil.

The findings of this study showed gaps in the equipment necessary for the reprocessing of dental items, including the following: the lack of autoclaves in good working condition in 1,328 (6.54%) units, while 8,406 (41.41%) had no sealers in good working condition, and 16,780 (82.66%) had no taps operating without contact. It is possible that there are difficulties in purchasing equipment, hiring technical professionals for equipment maintenance, and elaborating architectural projects when following the recommendations of sanitary legislations regarding the physical structure for units that provide dental care [4, 24]. The decisions related to the reprocessing of medical equipment/devices should be made by a Multidisciplinary Committee for Infection Prevention and Control and include professionals responsible for purchasing, reprocessing, maintenance, infection prevention and control, occupational safety, and final user of the instruments/devices [22]. We recommend the evaluation, continuous monitoring, and institution of validation protocols for the reprocessing of dental articles used in public services as a strategy to ensure the quality of the infection control process. Whenever possible, reprocessing should be performed in a specific environment that has the physical structure and qualified human resources to perform reprocessing [4, 5]. Problems in infrastructure of oral health services at PHC have been already been identified in Brazil and could impact the effectiveness and safety of dental care [25]. As a decentralized system, Brazilian National Health System [2], each municipality could organize its biomedical section or department, including their services/clinical engineers/technicians/outsources, to organize infrastructure locally. So, there is a variety of models of managing and organizing PHC structure in Brazil [25].

Although the data were collected before the onset of the COVID-19 pandemic, the situation of the lack of PPE for oral health professionals may have been worsened recently by the lack of new PPE [4–6]. One study has pointed out the high increase in costs and the difficulty in purchasing them [26]. Direct costs in biosafety before COVID-19 were R$0.84 per patient, R$6.69 per care shift, and R$3,413.94 per year [26]. With the COVID-19 pandemic, the costs increased to R$16.01 per patient, R$128.07 per shift, and R$32,657.96 per year [26]. This scenario can have negative repercussions on the planning of public oral health actions, because financial health resources were directed towards the purchase of biosafety inputs, besides limiting the population's access to oral health services in PHC [26]. Therefore, the rational use of PPE and the prioritization of care are crucial.

It is suggested that future national evaluations of PHC infection control incorporate questions regarding the biological monitoring of the reprocessing of dental instruments. Moreover, the study design did not allow for the association of factors related to failures in dental infection control. In this sense, future studies are necessary to identify factors associated with failures in the infection control process in public dental units of PHC.

The description of ICSS showed regional gaps and inequalities in infection control in those OHT in PHC in Brazil. The weakness in the infection control structure was related to the physical structure, equipment, supplies for infection control, and lack of PPI in sufficient quantity for OHT in PHC. The identification of these issues could help in policy-making and government funding towards primary dental health care to overcome the deficit in the structure and, as consequence, the quality of oral health services in PHC. PMAQ-AB has been an interesting model for evaluating and increasing the quality of PHC in Brazil. Unfortunately, this program has been discontinued in the recent years, with possible impacts in the future of the Brazilian National Health System [27].

## 5. Conclusions

Regional inequalities in the failures in infection control structures identified in PHC with OHT were related to the physical structure, equipment, and supplies used for infection control and the absence of PPE for OHT.

## Figures and Tables

**Table 1:** Descriptive analyses of infection control structure in 20,301^∗^ dental offices at PHC, Brazil, 2017-2018.

Variables (yes)	*n*	%
Good ventilation or air conditioning	18,796	92.6
Floor and walls with washables surfaces	15,791	77.8
Mold near sink	1,655	8.2
Tap without running water	507	2.5
Pungent sewage smell	347	1.7
Lack of water	400	2.0
Autoclave in use	18,973	93.5
Pack sealer in use	11,895	58.6
Nontouch tap	3,521	17.3
Sharp container	19,586	96.5
Rubber gloves for cleaning dental instruments	15,638	77.0
Materials/products for cleaning dental instruments and drills	18,128	89.3
Products for packaging dental instruments for sterilization	19,070	93.9
Personal protective equipment (PPE) in sufficient quantity	18,683	92.0

^∗^From 22,046 dental offices at PHC (URL: https://sage.saude.gov.br/) evaluated at the third cycle of PMAQ-AB, a number of 1,745 were excluded for the following reasons: unit had been disabled; the management did not authorize the assessment of the unit; the OHT did not authorize the assessment; the unit was being remodeled or expanded, and the team was not doing service anywhere else; the team did not join the PMAQ-AB; the OHT worked permanently elsewhere; the OHT worked with a specific population that was not available to the PMAQ-AB (remote area, penitentiary system, and mobile team). PPE: safety glasses, caps, procedure gloves, and masks in sufficient quantity.

**Table 2:** Factorial loads with Varimax rotation of the variables used for the development of the ICSS of public health units with OHT participants in the PMAQ-AB's third cycle, Brazil, 2017-2018.

Variable	Factor 1	Factor 2	Factor 3
Good ventilation	0.189	0.510	0.188
Floor and walls with washable surfaces	0.116	0.517	0.121
Mold near sink	0.572	0.335	0.064
Taps without running water	0.905	-0.082	0.097
Pungent sewage smell	0.732	0.277	0.002
Lack of water	0.769	0.084	0.148
Number of autoclaves in good working condition	0.164	0.074	0.618
Number of pack sealer in good working condition	0.048	-0.001	0.531
Sharp container	0.062	0.271	0.482
Rubber gloves for cleaning instruments	0.095	0.318	0.488
Products for cleaning dental instruments and drills	0.080	0.272	0.565
Products for packaging dental instruments for sterilization	0.095	0.027	0.913
Personal protective equipment (PPE)	0.126	0.281	0.482

PPE: safety glasses, caps, procedure gloves, and masks in sufficient quantity.

## Data Availability

The data used to support the findings of this study are available from the corresponding author upon request.
